# The delivery of new tuberculosis vaccines to people living with HIV – when to vaccinate?

**DOI:** 10.1186/s12879-025-11249-y

**Published:** 2025-07-01

**Authors:** Tom Sumner, Rebecca A. Clark, Tomos O. Prys-Jones, Roel Bakker, Gavin Churchyard, Richard G. White

**Affiliations:** 1 TB Modelling Group and TB Centre, London School of Hygiene and Tropical Medicine, Keppel Street, London, UK; 2 Centre for the Mathematical Modelling of Infectious Diseases, London School of Hygiene and Tropical Medicine, London, UK; 3 Department of Infectious Disease Epidemiology, London School of Hygiene and Tropical Medicine, London, UK; 4 KNCV Tuberculosis Foundation, The Hague, Netherlands; 5 The Aurum Institute, Johannesburg, 2041 South Africa; 6 School of Public Health, University of Witwatersrand, Johannesburg, South Africa; 7 Department of Medicine, Vanderbilt University, Nashville, TN USA

**Keywords:** Tuberculosis, Vaccines, HIV, Modeling, Anti-retroviral therapy

## Abstract

**Background:**

Tuberculosis (TB) remains a major cause of morbidity and mortality in people living with HIV (PLHIV). New TB vaccines may help reduce this burden. There is limited data on the response to new TB vaccines in PLHIV and how this may vary with levels of immunosuppression and anti-retroviral therapy (ART). The potential interaction between vaccine efficacy and ART raises questions about the optimum timing of vaccination against TB in PLHIV.

**Methods:**

Using a simple cumulative risk model, we compared the impact of different TB vaccination strategies for PLHIV. We compared the impact of vaccinating at linkage to HIV care, to the impact of vaccinating at ART initiation. We explored how the optimum timing of vaccination depends on characteristics of the vaccine and the ART program at an individual and population level.

**Results:**

For an individual, the optimum timing of vaccination against TB is at ART initiation unless the time to ART initiation is more than 6 months or if the reduction in vaccine efficacy when given prior to ART is small. At a population level, the proportion of PLHIV who initiate ART is a key determinate of the optimum strategy. If ART uptake is low, it would be better to vaccinate at linkage to HIV care, even if vaccine efficacy in ART naïve individuals is less than 50% of efficacy in individuals on ART.

**Conclusions:**

Our results suggest that the optimum timing of new TB vaccination for PLHIV will depend on the relative efficacy of vaccination in ART-naïve individuals vs. individuals on ART, and the uptake and timing of ART initiation. If vaccine efficacy is lower among ART-naïve individuals, improvements in HIV programs may help maximize the impact of new TB vaccines.

**Supplementary Information:**

The online version contains supplementary material available at 10.1186/s12879-025-11249-y.

## Background

Tuberculosis (TB) remains a major cause of morbidity and mortality in people living with HIV (PLHIV) [1]. New TB vaccines could play an important role in reducing the burden of TB in this population.

Bacillus Calmette-Guérin (BCG), the only currently licensed vaccine for TB, is widely used in infants and is effective in preventing severe forms of tuberculosis [2]. However, evidence suggests the efficacy of BCG may wane over time [3]. In addition, safety concerns remain over the use of BCG in PLHIV, especially in people not established on ART.

Several new TB vaccines, intended for use in adolescents and adults, are in development [4]. Trials have shown that these may be safe and immunogenic in PLHIV [5–9]. However, apart from one trial, there is no efficacy data in this population. The DarDar trial [10] found that 5 doses of an inactivated whole cell mycobacterial (*M. vaccae*) vaccine was associated with reductions in definite TB (a secondary endpoint of the trial) suggesting that TB vaccines may be effective in PLHIV. There is also little data on how levels of immunosuppression and anti-retroviral therapy (ART) may affect TB vaccine performance [11].

Evidence from other vaccine preventable diseases suggests that vaccination of individuals established on ART induces a greater immune response compared to vaccination of ART naive individuals [12, 13]. A review of recent studies examining the safety, immunogenicity and effectiveness of anti-SARS-CoV-2 vaccines suggests that PLHIV with viral suppression exhibited similar immune responses to people without HIV while PLHIV who had detectable viremia had lower immune responses [14]. In addition, studies of measles vaccination in children with HIV suggest that children established on ART have a more durable response to vaccination than those vaccinated before initiating therapy [15]. Studies have also shown that initiating ART does not restore vaccine induced immunity to measles when started after vaccination [16]. However, any potential improvement in vaccine efficacy obtained by delaying vaccination until ART initiation has to be balanced against other factors [13] including the risk of infection during this delay and that individuals who do not start ART may miss out on vaccination.

For any newly licensed TB vaccine there will be similar questions around the timing of targeted vaccination for PLHIV relative to ART initiation. Should you vaccinate PLHIV as soon as they are diagnosed and linked to HIV care? Or wait until they initiate ART? And how does this depend on the vaccine characteristics, the risk of TB disease and the uptake of ART?

In this work, we used a simple cumulative risk model to compare the impact of different vaccination strategies in PLHIV while varying several unknown vaccine and ART programme characteristics.

The results could help inform future vaccine roll-out plans in PLHIV as and when new TB vaccines indicated for PLHIV are licensed.

## Methods

### Cumulative risk model

The model population consisted of PLHIV from the time that they are diagnosed with HIV (*t* = 0) and linked to HIV care.

To quantify the impact of different TB vaccination strategies, we defined expressions for the probability of an individual remaining TB free, *s*(*t*), by time since linkage to HIV care (*t*). The cumulative risk of incident TB disease was given by 1-*s*(*t*).

In the absence of vaccination or ART, the per unit time risk of incident TB is *x*. Individuals start ART at time *t*_*ART*_. The hazard ratio for incident TB when on ART compared to ART naïve individuals is *A*.

We explored three different scenarios for the timing of vaccination against TB: (1) no vaccination; (2) vaccination at the time of linkage to HIV care; (3) vaccination at the time of ART initiation.

In this population of PLHIV we assumed the efficacy of vaccination was highest when given to individuals on ART *(v)*. We assumed that vaccine efficacy was reduced if given prior to ART initiation. We define this reduction in efficacy via the relative efficacy in ART-naïve individuals compared to ART-experienced individuals (*RE*_*v*_ < 1). We also assumed that for an individual vaccinated prior to ART initiation, vaccine efficacy would not improve after ART initiation.

We also make a number of simplifying assumptions. We assumed that all individuals did not have TB at the start of the simulation (t = 0). The risk of TB in the absence of vaccination or ART was assumed to be constant and to change instantaneously upon vaccination or ART initiation. We assumed that the duration of protection of vaccination was greater than the time horizon modelled, that the duration of protection of vaccination against TB does not depend on ART status, and that there is no non-TB associated mortality over the time horizon modelled. Tuberculosis preventive therapy (TPT) is known to reduce the risk of TB in PLHIV and is recommended by the World Health Organisation (WHO) for all PLHIV. However, given uncertainties about how TPT and vaccines may be used in combination, and to allow us to focus our analysis on the interaction between vaccines and ART, we did not explicitly model (TPT).

The expressions are shown in Table 1.

**Table 1:** Expressions for the probability of remaining TB free by vaccination and ART status. S(*t)* is the probability of remaining TB free at time *t*; *x* is the per unit time risk of TB in the absence of vaccine or ART; *A* is the hazard ratio for TB when on ART (compared to ART Naive individuals); *v* is the efficacy of vaccination against TB in individuals on ART; *RE*_*v*_ is the relative efficacy of vaccination against TB in individuals not on ART (compared to people on ART); *t*_*ART*_ is the time to ART initiation (from time of linkage to HIV care)

Timing of vaccination	Probability of remaining TB free at time t	
None	$\:S\left(t\right)=\text{e}\text{x}\text{p}(-xt)\:$	for 0 ≤ t < t_ART_
	$\:S\left(t\right)=\text{exp}\left(-x{t}_{ART}\right)\text{e}\text{x}\text{p}(-xA\left(t-{t}_{ART}\right))$	for t ≥ t_ART_
At linkage to HIV care	$\:S\left(t\right)=\text{e}\text{x}\text{p}(-x\left(1-v{RE}_{v}\right)t)\:$	for 0 ≤ t < t_ART_
	$\:S\left(t\right)=\text{exp}\left(-x\left(1-v{RE}_{v}\right){t}_{ART}\right)\text{e}\text{x}\text{p}(-xA\left(1-v{RE}_{v}\right)\left(t-{t}_{ART}\right))$	for t ≥ t_ART_
At ART initiation	$\:S\left(t\right)=\text{e}\text{x}\text{p}(-xt)$	for 0 ≤ t < t_ART_
	$\:S\left(t\right)=\text{exp}\left(-x{t}_{ART}\right)\text{e}\text{x}\text{p}(-xA(1-v\left)\left(t-{t}_{ART}\right)\right)$	for t ≥ t_ART_

### Individual simulations

To explore the effect of the timing of vaccination at the individual level, we simulated the cumulative risk of incident TB for individuals who initiate ART for the 3 scenarios of vaccination timing listed in Table 1 (no vaccination; vaccination at linkage to HIV care; vaccination at ART initiation).

We explored how the risk of incident TB in these 3 vaccination scenarios depended on the relative efficacy of vaccination against TB in individuals not on ART compared to those on ART (*RE*_*v*_), and the time to ART initiation (from time of linkage to HIV care) (*t*_*ART*_).

In our main analysis we assumed that the annual risk of TB in the absence of ART (*x*) was 1%. We assumed that the hazard ratio for TB when on ART (compared to ART naïve individuals) (*A*) was 0.35 [17]. We assumed the efficacy of vaccination against TB in individuals on ART (*v*) was 50%, based on the minimum required efficacy specified in the World Health Organization preferred product characteristics for new TB vaccines [18]. We carried out sensitivity analysis to understand how the results depend on these assumptions.

### Population simulations

To explore the effect of the timing of vaccination at the population level, we simulated the cumulative incidence of TB in populations with given distributions of ART uptake and time to ART initiation. This was done by taking weighted averages of the individual risk profiles based on the composition of the population over time.

In our primary analysis, we based the time to ART distribution on data from the HPTN 071 (PopART) Study [19]. HPTN 071 was a community randomized clinical trial, which evaluated a combination HIV prevention package. Participants were offered point of care finger prick HIV testing, delivered by community HIV care providers with people testing HIV positive referred to local clinics for linkage to HIV care. In the study, ART was offered immediately irrespective of CD4 count, consistent with current WHO guidelines. In this work, we used estimates of the time from first linkage to HIV services (i.e. first clinic attendance) to ART initiation. In this population 64% of people had initiated ART within 1 month and 94% within 12 months (see Fig. 3A). This figure is consistent with a recent systematic review that found a prevalence of delayed ART initiation of 36% [20]. We assumed that people who had not started ART within 12 months would never start.

To explore how the results depended on the uptake of ART and the time to ART initiation we then simulated alternative distributions in which we varied the maximum proportion starting ART by 12 months, and the time taken for 50% of the population to initiate ART. Figure 4A shows examples of the distributions used.

## Results

**Fig. 1:**
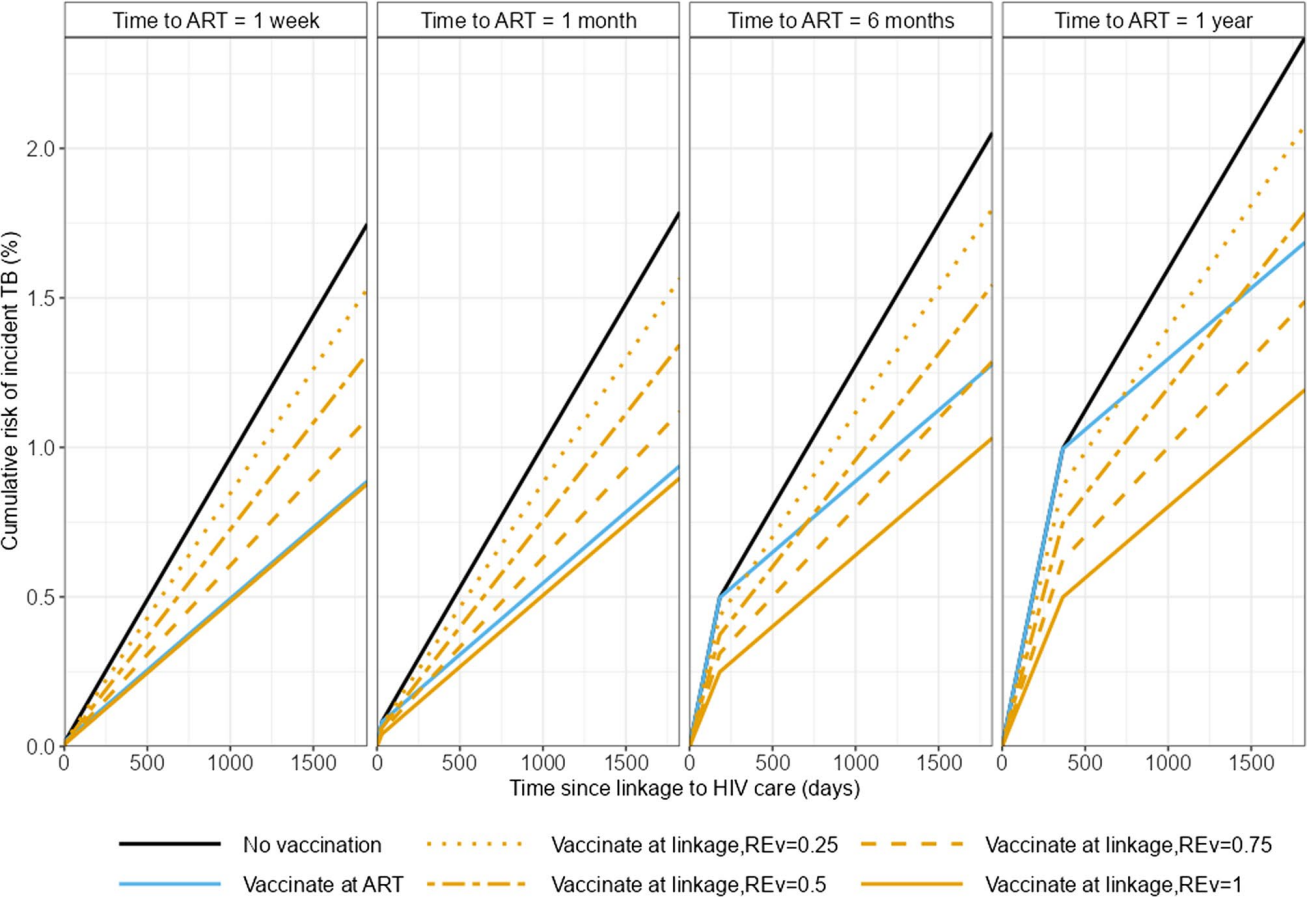
Cumulative risk of incident TB in individuals by time since linkage to HIV care. Colours show the different scenarios for the timing of vaccination. Line types show different relative vaccine efficacy in the absence of ART (compared to when on ART) (*RE*_*v*_). Columns show different times to ART initiation (*t*_*ART*_)

Figure 1 shows the cumulative risk of incident TB in an individual from their time since linkage to HIV care.

If the vaccine efficacy in people not on ART is the same as in people on ART (Relative vaccine efficacy = 1; solid lines in Fig. 1) the cumulative risk of TB is lower if vaccination is given at linkage to HIV care (solid orange lines) than if vaccination is delayed until ART initiation (solid blue lines). How much lower depends on the time to ART initiation. With short times to ART initiation the difference is minimal (compare solid orange and blue lines in left panel of Fig. 1) whereas with time to ART initiation of 1 year there are substantial differences in the risk of incident TB (compare solid orange and blue lines in right panel of Fig. 1).

If the vaccine is less effective when given to people not on ART compared to people on ART (Relative vaccine efficacy < 1), and the time to ART initiation is short, it is better to wait until individuals start ART before vaccinating against TB (compare the non-solid orange lines to the blue line in the left panel of Fig. 1). This is because the increase in vaccine efficacy achieved by delaying vaccination outweighs the increased risk of disease over the short time period prior to ART initiation (and vaccination). As the time to ART initiation increases, the best strategy varies depending on the time to ART initiation and the relative vaccine efficacy. If the time to ART initiation is sufficiently long and the relative efficacy is high, it may be preferable to vaccinate at linkage to HIV care rather than wait until ART initiation.

Figure 1 also shows that the “best” choice for the timing of vaccination depends on the time horizon considered. An example of this is shown in the right hand panel of Fig. 1 comparing vaccination at ART initiation (blue line) to vaccination at linkage to HIV care with a relative vaccine efficacy of 0.25 (dotted orange line). In the short term, the cumulative risk of TB is lowest when vaccination is given at linkage to HIV care (dotted orange line) but over the longer term the cumulative risk of TB is lower when vaccination is given at ART initiation (blue line). This is because in the short term individuals are not protected if vaccination is delayed until ART initiation; in the longer term the increased efficacy of vaccination when given after ART reduces the risk of TB.

In the remainder of this analysis, we focus on the long term outcomes (5 years; 1825 days) after linkage to HIV care.

**Fig. 2:**
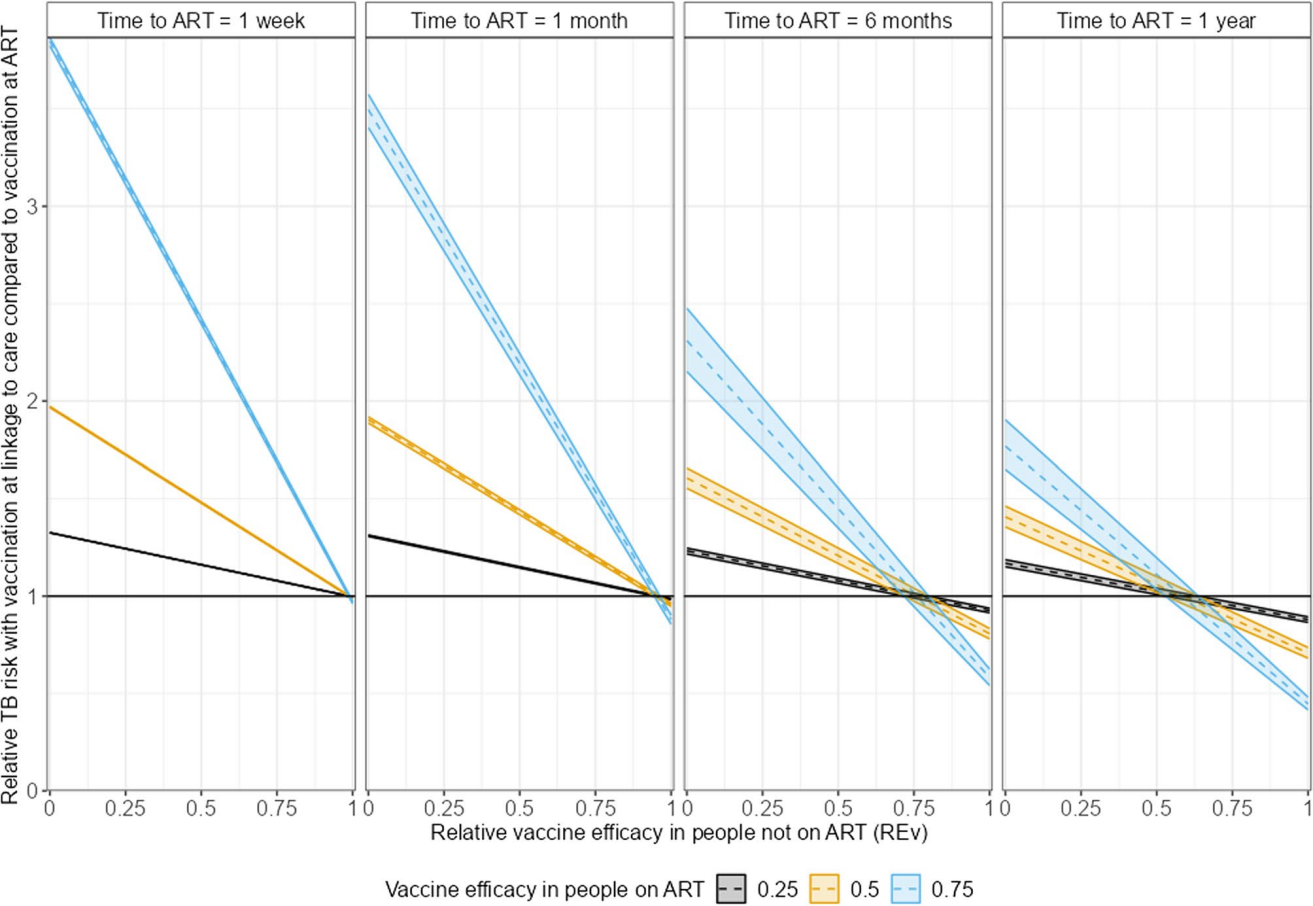
Relative risk of incident TB with vaccination at linkage to HIV care (compared to vaccination at ART initiation) versus relative vaccine efficacy in people not on ART (*RE*_*v*_). Colours show different vaccine efficacy in people on ART (*v*). Shaded regions show variability due to uncertainty in the hazard ratio for TB when on ART (compared to ART naive individuals) (*A*). Columns show different times to ART initiation (*t*_*ART*_). All results are for a time horizon of 5 years from linkage to HIV care

Figure 2 shows the relative risk of incident TB with vaccination at linkage to HIV care, compared to when vaccination is given at ART initiation, as a function of the relative vaccine efficacy in people not on ART compared to those on ART (*RE*_*v*_). If this value is greater than 1 (above the black horizontal lines), it is better to wait until ART initiation to carry out vaccination. The point at which the line crosses 1 shows the threshold value of *RE*_*v*_ at which the optimum timing of vaccination changes.

The panels show results for different times to ART initiation. Both the relative difference between vaccination timing scenarios and the threshold value of *RE*_*v*_ depend on the time to ART initiation. The threshold value of *RE*_*v*_ at which it is better to vaccinate at linkage to HIV care reduces as the time to ART initiation increases.

The colours show results for different values of vaccine efficacy in people on ART (*v*). The relative difference between scenarios depends on *v* but the threshold value of *RE*_*v*_ does not.

The shaded areas show the variability in results across a range of values for the hazard ratio for TB while on ART (*A*) from 0.28 to 0.44 [17]. This shows that both the relative difference and the threshold are largely independent of the effectiveness of ART.

In the appendix we also show, analytically and graphically, that the relative difference between scenarios is largely independent of the annual risk of TB in the absence of ART (*x*) and that the threshold value of RE_v_ is independent of *x*.

The results above assume that for an individual vaccinated prior to ART initiation, vaccine efficacy would not improve after ART initiation. If instead vaccine efficacy is dependent on current ART status (i.e., in an individual vaccinated prior to ART initiation, vaccine efficacy would increase after ART initiation to the same level as in someone vaccinated after ART initiation) then we find that it would always be better to vaccinate at linkage to HIV care. Figure A2 in the appendix shows the results of this sensitivity analysis.

**Fig. 3:**
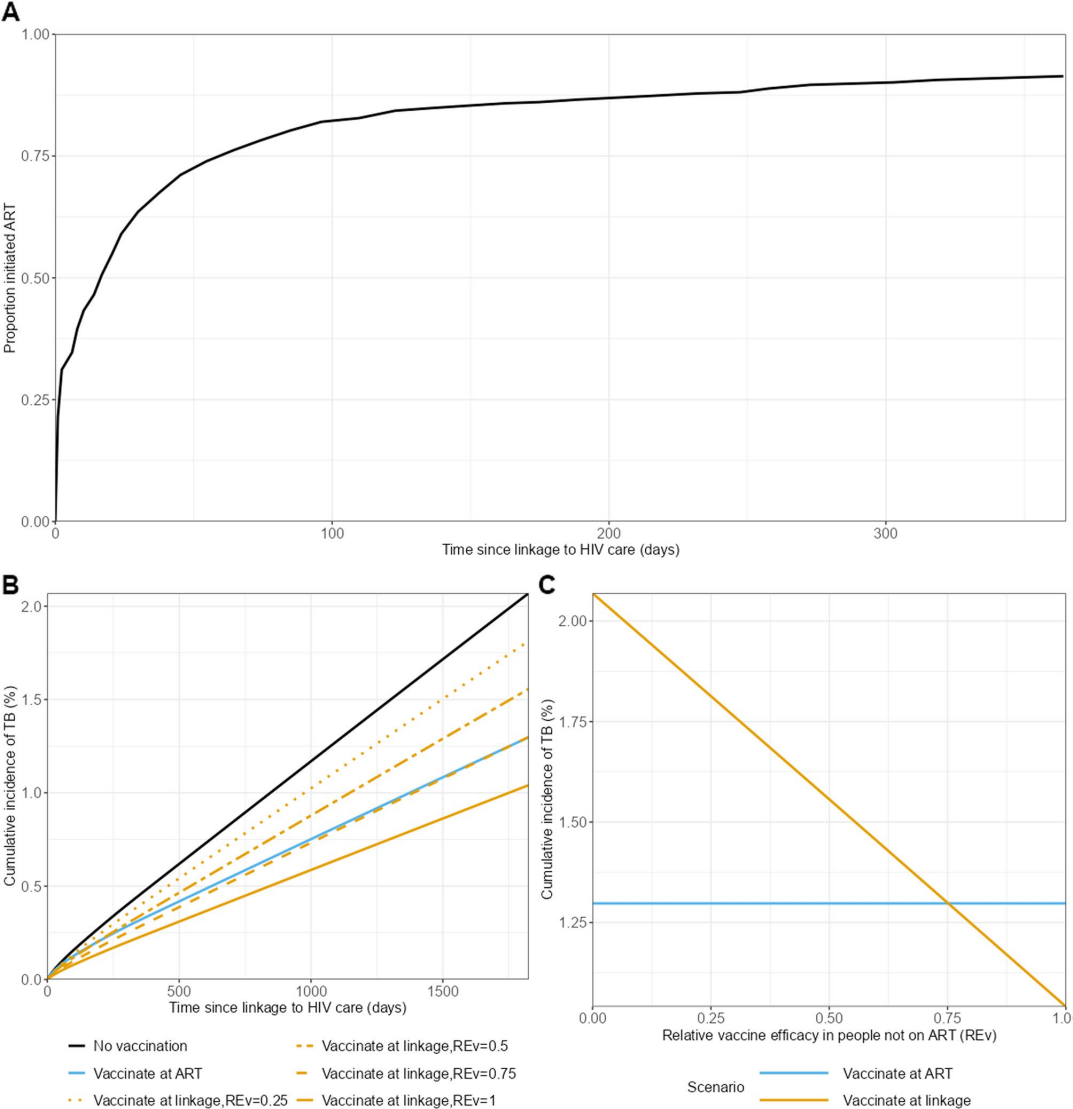
Results of population analysis with 94% ART coverage (based on HPTN 071 cohort). **A** Proportion of the population who have initiated ART by time from linkage to HIV care. **B** Cumulative incidence of TB vs. time since linkage to HIV care. **C** Cumulative incidence of TB at 5 years since linkage to HIV care versus relative vaccine efficacy in the absence of ART (compared to when on ART) (*RE*_*v*_). Colors show the vaccination timing scenarios; line types show different values for the relative vaccine efficacy in the absence of ART (compared to when on ART) (*RE*_*v*_)

Figure 3 shows the results of the population simulations based on time to ART data from the HPTN 071 (PopART) Study [19] in which 64% of people initiated ART within 1 month and 94% within 12 months since linkage to HIV care (Fig. 3A).

Figure 3B shows the cumulative incidence of TB in the population by time since linkage to HIV care. The black line shows the incidence in the absence of vaccination, the blue line shows vaccination at ART initiation and the orange lines show vaccination at linkage to HIV care for different values of the relative vaccine efficacy in people not on ART (compared to people on ART) (*RE*_*v*_). This shows that, as expected, when vaccination is given at linkage to HIV care, the incidence of TB decreases as *RE*_*v*_ increases.

Figure 3C shows the cumulative incidence at 5 years since linkage to HIV care as a function of the relative vaccine efficacy in people not on ART (compared to people on ART) (*RE*_*v*_). The blue line shows vaccination at ART initiation (which does not depend on *RE*_*v*_), the orange line shows vaccination at linkage to care. For this example population, it is better to vaccinate at ART initiation (lower TB incidence) for values of REv below 0.75; above this value it is better to vaccinate at linkage to HIV care

**Fig. 4:**
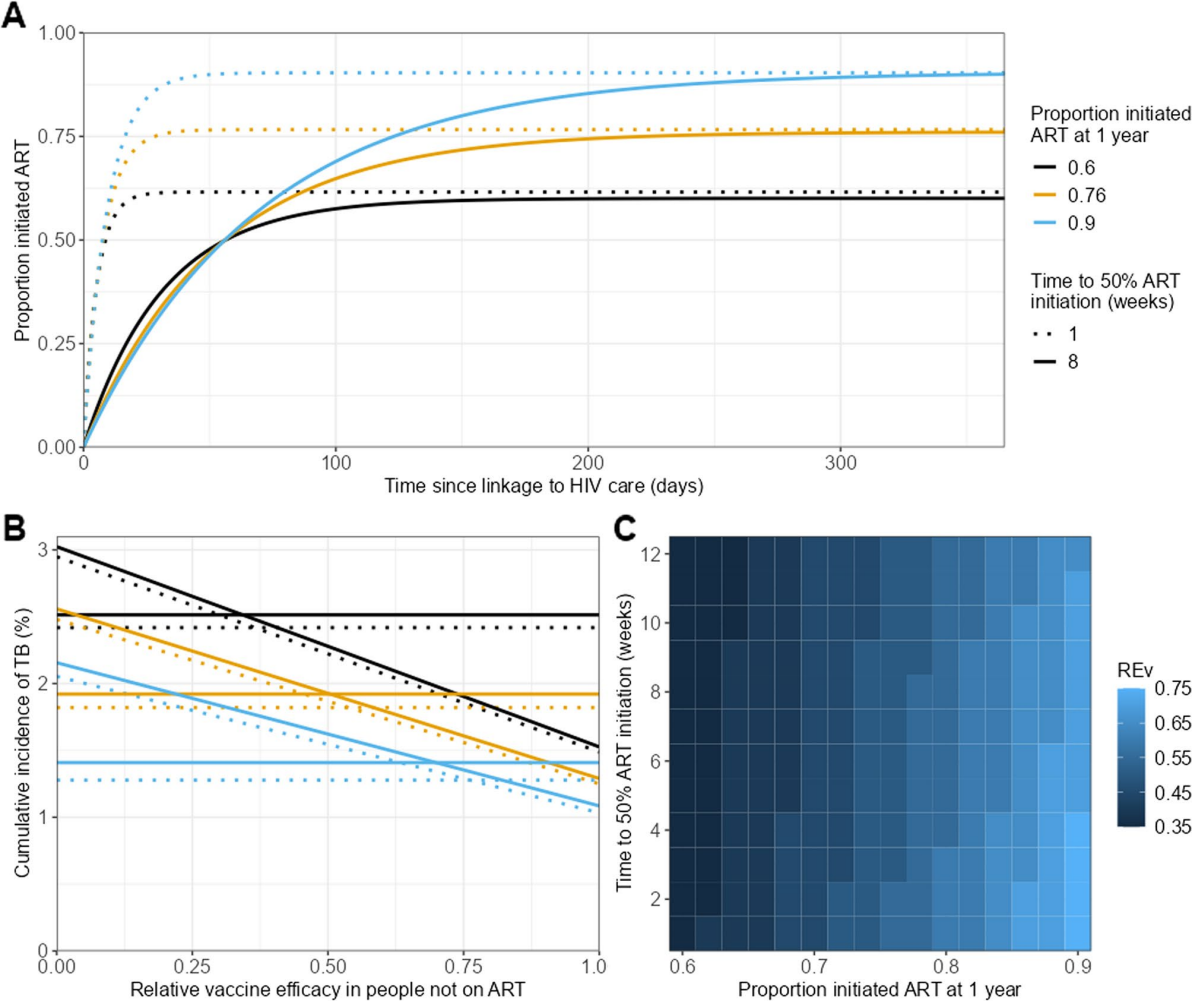
Results of hypothetical population analysis. **A** Example distributions of time from linkage to HIV care to ART initiation. **B** Cumulative incidence of TB at 5 years since linkage to HIV care versus relative vaccine efficacy in the absence of ART (compared to when on ART) (*RE*_*v*_) for different hypothetical populations. Colors show the proportion of the population initiated on ART at 1 year, line types show the time to 50% ART initiation (in weeks). Horizontal lines showing the incidence when vaccination is given at ART initiation, declining lines show the incidence when vaccination is given at linkage to HIV care. **C** Threshold value of the relative vaccine efficacy in those not on ART (*RE*_*v*_) as a function of proportion initiating ART after 1 year and time to 50% ART initiation

Figure 4 shows how the population simulation results depend on the ART uptake and time to ART distribution. We simulated results for populations in which the proportion who start ART varied between 60 and 90% and the time to 50% ART initiation varied between 1 and 12 weeks. Figure 4A shows example time to ART distributions with 60, 75 and 90% coverage, and times to 50% coverage of 1 or 8 weeks.

Figure 4B shows the cumulative TB incidence in the population after 5 years against *RE*_*v*_ for each of the populations shown in Fig. 4A. The horizontal lines show the incidence when vaccination is carried out at ART initiation. The declining lines show the incidence when vaccination is carried out at linkage to HIV care. As in Fig. 3C, the point where the lines cross shows the threshold *RE*_*v*_ value above which it is better (i.e. TB incidence is lower) when vaccination is carried out at linkage to HIV care. For example, with ART coverage of 75% (orange lines) it is better to vaccinate at linkage to HIV care if *RE*_*v*_ is greater than 50%.

Figure 4C shows how the threshold value of relative vaccine efficacy in the absence of ART (compared to when on ART) (*RE*_*v*_) above which it is better to vaccinate at linkage to HIV care varies as the proportion who start ART in one year (x-axis) and the time to 50% ART initiation (y-axis) are varied.

Both Fig. 4B and C illustrate that the threshold value of *RE*_*v*_ above which it is better to vaccinate at linkage to HIV care increases when the proportion initiating ART is increased (strong dependence) or when the average time to ART initiation is reduced (weak dependence).

## Discussion

In this paper, we used a simple cumulative risk model to explore different strategies for the use of new TB vaccines in PLHIV. We estimated how timing of vaccination (at linkage to HIV care or at ART initiation) affected the risk of incident TB and how this risk depended on several unknown parameters.

Our results suggest that for an individual it is better to wait to vaccinate at ART initiation unless the time to ART initiation is long and/or if the reduction in vaccine efficacy when given prior to ART is small. For example, with a time to ART initiation of 1 month the reduction in vaccine efficacy must be less than 10% to favor vaccination at linkage to HIV care. Similarly with a delay of 6 months the reduction in efficacy must be less than 25%.

When considering vaccination at a population level, our results illustrate how the choice will depend on the performance of the ART program. If the ART program is weak and therefore a smaller proportion of PLHIV initiate ART, it is better to vaccinate at linkage to HIV care at the cost of reduced vaccine efficacy (lower *RE*_*v*_) to provide protection to those who would not start ART (and therefore miss out on vaccination offered at ART initiation). If the ART program is strong and a higher proportion of people start ART, the relative vaccine efficacy in those not on ART (*RE*_*v*_) must be higher to favour vaccination at linkage to HIV care.

These results make intuitive sense. This paper is the first attempt to quantify the vaccine characteristics and ART coverage for which one vaccination scenario could be favored over another. It also illustrates how sensitive the findings are to other assumptions. In particular, we found that the threshold value of the relative vaccine efficacy in those not on ART (RE_V_) at which the optimal choice of the timing of vaccination changes was largely independent of the vaccine efficacy in people on ART (*v*), the protective effect of ART against incident TB (*A*) and the risk of incident TB (*x*). These findings may be useful when considering the generalizability of vaccination strategies for PLHIV to different settings or populations.

In our primary analysis we assumed that TB vaccine efficacy depended on the ART status at the time of vaccination, that is, in an individual vaccinated prior to ART initiation vaccine efficacy would not improve after ART initiation. If vaccine efficacy did increase after ART initiation to the same level as in someone vaccinated while on ART then it would always be better to vaccinate at linkage to HIV care. In this case all individuals would experience the same vaccine efficacy while on ART and those vaccinated prior to ART would experience some additional time period of protection due to vaccination. If efficacy was increased to some intermediate level after ART initiation then we would expect the results to lie somewhere between these 2 limiting cases. It is currently unknown how ART initiation may affect the efficacy of prior vaccination for TB. Evidence from measles vaccination suggests that ART did not restore vaccine-induced immunity when started after vaccination [15, 16]. However, some vaccines with strong T-cell components may produce increased responses following immune restoration due to ART.

Our model is very simple and makes a number of assumptions that could be refined. We assumed a constant risk of TB at linkage to HIV care (in the absence of ART or vaccination). In reality, the risk of TB may vary considerably between individuals depending on their level of immunosuppression at the time of HIV diagnosis. In addition, the risk of TB may increase between linkage to care and ART initiation due to continued declines in CD4 counts. Including this variable and increasing risk may favour vaccination at linkage to HIV care as the cumulative risk of TB prior to ART initiation would be increased. However due to the relatively short time period between linkage to HIV care and ART initiation (at most 1 year in our analysis) we believe the effects of this assumption on our results would be small. Data from ART-naïve individuals’ suggests that annual declines in CD4 count range between 22 and 100 cells/ul [21] and that the risk of TB increases by 1.43 fold per 100 decline in CD4 count [22]. Together these suggest the increase in risk between linkage to care and ART initiation would be modest.

We also assumed instantaneous changes in TB risk following ART initiation or vaccination. In reality the improvement in immune response, particularly after ART initiation will be more gradual and maximal vaccine efficacy may only occur weeks after completing a full vaccination schedule, which may require multiple doses over two to three months. Our model also assumed that the duration of vaccine protection is greater than the time horizon of our model and does not vary by ART status. A reduction in duration of protection in ART-naïve individuals would likely favour delaying vaccination until ART initiation. It is also possible that vaccine efficacy would vary by level of immunosuppression, not just by ART status as assumed in our model. However there is limited data on how immune responses to new vaccines vary by CD4 count as most studies have been conducted in individuals with CD4 counts exceeding 350 cells/mm^3^. Further work could explore the importance of this assumption.

Our model did not explicitly account for TPT. TPT is known to reduce the risk of TB but how it may be used in combination with new vaccines is unknown. Increased uptake of TPT and the development of new TPT regimens would reduce the risk of TB in PLHIV and potentially reduce the incremental benefit of new vaccines and the relative benefit of delayed versus early vaccination. It is also possible that new highly efficacious vaccines may replace TPT regimens if they are more acceptable to individuals due to the “one off” nature of a vaccine and the potential to remove the side-effects associated with TPT drug regimens.

We only considered scenarios involving a single vaccination delivered before or after ART initiation. Alternative strategies could involve vaccinating individuals at linkage to HIV care and providing a booster at ART initiation. A 2 vaccine schedule would likely be more effective than either single vaccine scenario considered in this paper but involves greater logistical challenges and increased costs. Future modelling could explore the impact and cost-effectiveness of one versus 2 vaccine strategies once data on the efficacy of specific vaccine candidates in PLHIV becomes available.

Finally, our analysis is largely theoretical and has not been calibrated to represent any real population. As such the results should not be used to directly inform vaccination strategies in any specific setting. Our finding that the results were robust to the assumed risk of TB suggest our broad findings may be generalizable, however population specific data on TB risk, CD4 count distributions and ART initiation would be needed to refine our model for real-world prediction.

## Conclusions

In conclusion, our analysis illustrates that the optimum use of targeting new TB vaccines in PLHIV depends on several factors including characteristics of the TB vaccine itself and the performance of the HIV program in the setting where TB vaccines would be used. Our results suggest that, if vaccine efficacy is reduced in ART-naïve individuals, improvements in HIV programs may help maximize TB vaccine impact. Our model could be further refined as more data on vaccine performance in PLHIV becomes available.

## Supplementary Information

Supplementary Material 1.

## Data Availability

All materials used in the analysis are available at: https://github.com/tomsumner/Timing_of_vaccination.

## Abbreviations

ART: Anti-retroviral therapy
BCG: Bacillus Calmette-Guérin
PLHIV: People living with HIV
TB: Tuberculosis
TPT: Tuberculosis preventive therapy
WHO: World Health Organisation
