# Supplementary material for: The delivery of new tuberculosis vaccines to people living with HIV – when to vaccinate?
Source: BMC Infect Dis. 2025 Jul 1;25:878. doi: 10.1186/s12879-025-11249-y (PMC12217277; doi:10.1186/s12879-025-11249-y)
Supplement: Supplementary file 1 — Supplementary Material 1. [file 12879_2025_11249_MOESM1_ESM.docx]

**Appendix**

**Analytical results**

Table A1 reproduces table 1 from the main text and shows the expression for the probability of remaining TB free by time since linkage to HIV care.

| **Timing of vaccination** | **Probability of remaining TB free at time t** |  |
| --- | --- | --- |
| None | $S\left( t \right)=exp(-xt)$ | for 0 ≤ t < t_ART_ |
|  | $S\left( t \right)=\exp\left( -xt_{ART} \right)exp(-xA\left( t-t_{ART} \right))$ | for t ≥ t_ART_ |
| At linkage to HIV care | $S\left( t \right)=exp(-x\left( 1-v{RE}_{v} \right)t)$ | for 0 ≤ t < t_ART_ |
|  | $S\left( t \right)=\exp\left( -x\left( 1-v{RE}_{v} \right)t_{ART} \right)exp(-xA\left( 1-v{RE}_{v} \right)\left( t-t_{ART} \right))$ | for t ≥ t_ART_ |
| At ART initiation | $S\left( t \right)=exp(-xt)$ | for 0 ≤ t < t_ART_ |
|  | $S\left( t \right)=\exp\left( -xt_{ART} \right)exp(-xA(1-v)\left( t-t_{ART} \right))$ | for t ≥ t_ART_ |

**Table A1. Expressions for the probability of remaining TB free by vaccination and ART status.** S(*t)* is the probability of remaining TB free at time *t*; *x* is the per unit time risk of TB in the absence of vaccine or ART; *A* is the hazard ratio for TB when on ART (compared to ART naive individuals); *v* is the efficacy of vaccination against TB in individuals on ART; *RE_v_* is the relative efficacy of vaccination against TB in individuals not on ART (compared to people on ART); *t_ART_* is the time to ART initiation (from time of linkage to HIV care).

From the expressions in table A1 we can calculate the ratio of the proportion TB free when vaccine is given at linkage to HIV care compared to when vaccine is given at ART initiation.

Prior to ART initiation (t < t_ART_) the ratio is given by:

$e^{xtvRE_{v}}$ (1)

As $xtvRE_{v}$ is always > 0, the ratio is always > 1. In other words, prior to ART initiation a greater proportion are TB free (meaning the cumulative incidence of TB is lower) if they are vaccinated at linkage to care than if vaccine is delayed until ART initiation. This is logical as individuals have additional protection due to vaccination in that case.

Post ART initiation (*t* > *t_ART_*) the ratio is given by:

$e^{x\left( t_{ART}+A\left( 1-v \right)\left( t-t_{ART} \right)-\left( 1-vRE_{v} \right)\left( t_{ART}+A\left( t-t_{ART} \right) \right) \right)}$ (2)

As the risk of TB in the absence of vaccine or ART (*x)* is typically small (<< 1) and the ratio is proportional to exp(*x*) changes in *x* do not significantly change the ratio post ART initiation.

From expression (2) we can derive the following inequality:

$\frac{t_{ART}\left( A\left( 1-RE_{v} \right)+RE_{v} \right)}{tA\left( 1-RE_{v} \right)}>1$ (3)

If this inequality is satisfied, then post ART initiation, a greater proportion are TB free (meaning the cumulative incidence of TB is lower) if vaccination is given at linkage to care than if it is delayed until ART initiation.

This inequality includes four variables; the time of interest (*t*); the time to ART initiation (*t_ART_*); the hazard ratio for TB when on ART (*A*); the relative efficacy of vaccination against TB in individuals not on ART (*RE_v_*). It does not depend on the baseline TB incidence (*x*) or the vaccine efficacy in people on ART (*v*).

**Additional numerical results**

Figure A1 shows the relative risk of incident TB with vaccination at linkage to HIV care compared to when vaccination is given at ART initiation as a function of the relative vaccine efficacy in people not on ART compared to those on ART (*RE_v_*). This is similar to figure 2 in the main text but shows results for different values of the TB risk in the absence of vaccine or ART (*x*) (shown by different line types). This confirms the analytical results that: i) the ratio of TB risk between the vaccine timing scenarios is not significantly altered by the risk of TB in the absence of vaccine or ART (*x*); ii) the threshold does not depend on the risk of TB in the absence of vaccine or ART (*x*) or the vaccine efficacy in people on ART (*v*).


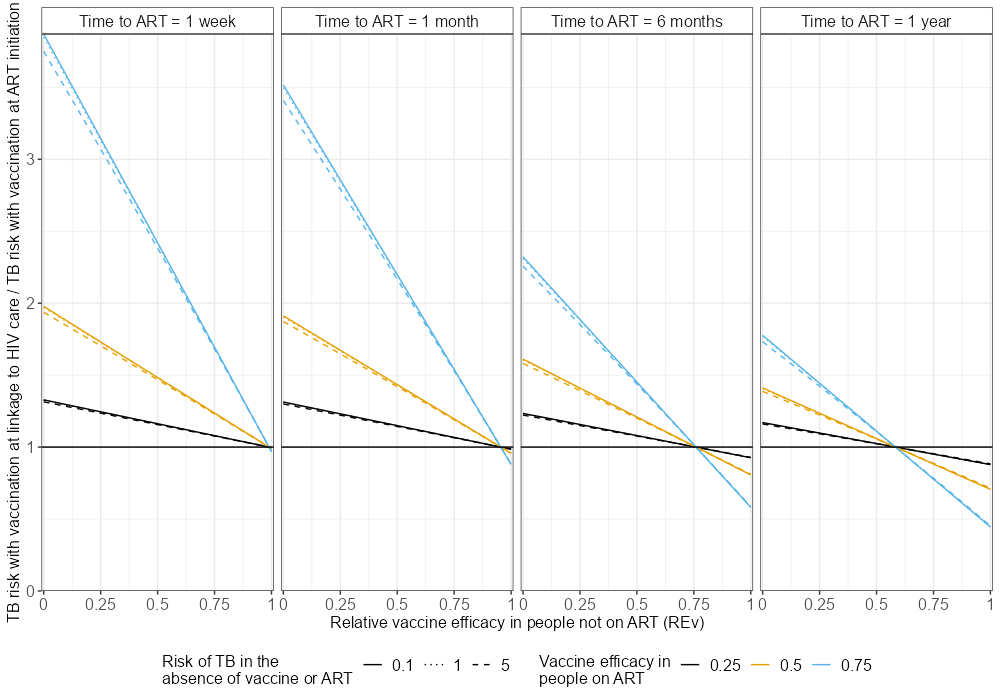


**Figure A1. Relative risk of incident TB with vaccination at linkage to HIV care (compared to vaccination at ART initiation) versus relative vaccine efficacy in people not on ART.** Colours show different vaccine efficacy in people on ART. Line types show different risk of TB in the absence of vaccine or ART. Columns show different times to ART initiation. All results are for a time horizon of 5 years from linkage to HIV care.

Figure A2 shows the results when we assume that the efficacy of vaccination given to an ART-naïve individual is increased when they start ART. In this case it is better to vaccinate at linkage to HIV care for any value of *RE_v_* or time to ART initiation.


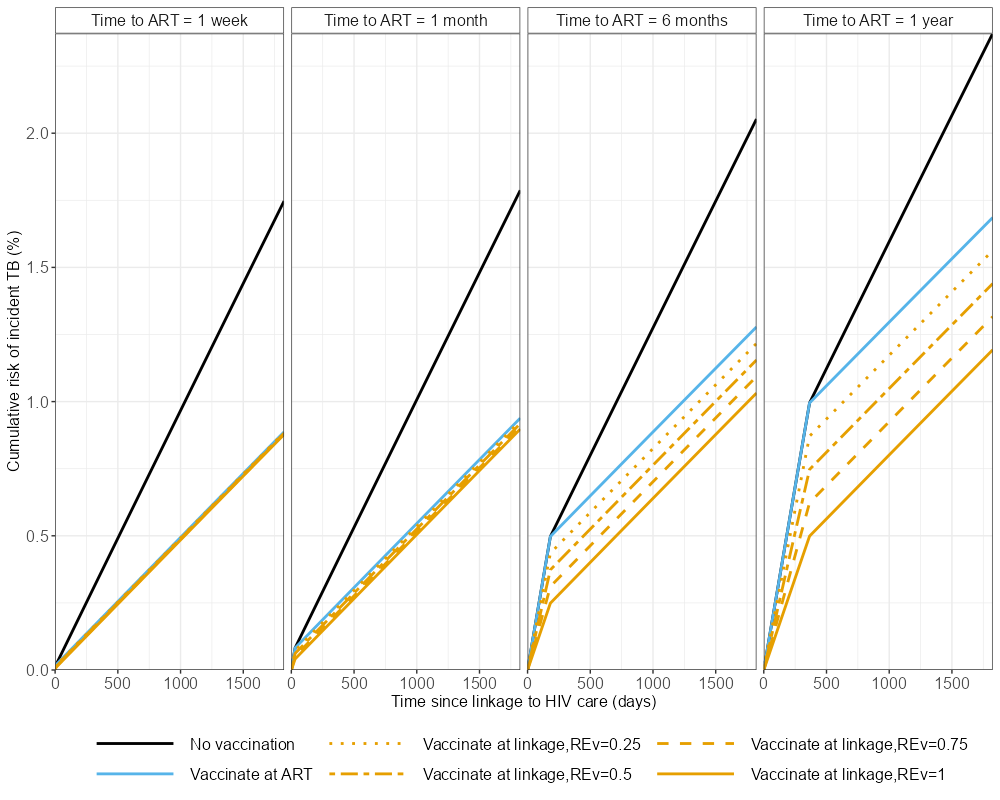


**Figure A2. Cumulative risk of incident TB in individuals by time since linkage to HIV care assuming that vaccine efficacy increases once on individual initiates ART.** Colours show the different scenarios for the timing of vaccination. Line types show different relative vaccine efficacy in the absence of ART (compared to when on ART) (*RE_v_*). Columns show different times to ART initiation (*t_ART_*).
